# The Changing Landscape of Acute Kidney Injury in Pregnancy from an Obstetrics Perspective

**DOI:** 10.3390/jcm8091396

**Published:** 2019-09-06

**Authors:** Angela Vinturache, Joyce Popoola, Ingrid Watt-Coote

**Affiliations:** 1 Department of Obstetrics & Gynaecology, Oxford University Hospitals NHS Foundation Trust, Oxford OX3 9DU, UK; 2 Department of Obstetrics & Gynaecology, St. George’s University Hospital NHS Foundation Trust, London SW17 0QT, UK; 3 Department of Nephrology and Transplantation, St. George’s University Hospital NHS Foundation Trust, London SW17 0QT, UK

**Keywords:** pregnancy, kidney, physiology of pregnancy, acute kidney injury, pregnancy-related acute kidney injury

## Abstract

Pregnancy-related acute kidney injury (PR-AKI) is a heterogeneous disorder with multiple aetiologies that can occur at any time throughout pregnancy and the post-partum period. PR-AKI is an important obstetric complication that is associated with significant maternal and foetal morbidity and mortality. Although there has been an overall decline in the incidence of PR-AKI worldwide, a recent shift in the occurrence of this disease has been reported. Following improvements in obstetric care, PR-AKI incidence has been reduced in developing countries, whereas an increase in PR-AKI incidence has been reported in developed countries. Awareness of the physiological adaptations of the renal system is essential for the diagnosis and management of kidney impairment in pregnancy. In this review we scrutinize the factors that have contributed to the changing epidemiology of PR-AKI and discuss challenges in the diagnosis and management of acute kidney injury (AKI) in pregnancy from an obstetrics perspective. Thereafter we provide brief discussions on the diagnostic approach of certain PR-AKI aetiologies and summarize key therapeutic measures.

## 1. Introduction

The ability of the body to adapt to the physiologic alterations of pregnancy is attenuated in women with kidney diseases or systemic diseases affecting the kidney. A variety of disorders cause acute kidney injury (AKI) in early or late pregnancy, with pre-eclampsia and pregnancy-induced hypertensive disorders being the most common etiology [1]. In turn, pregnancy-related hypertensive disorders and AKI are linked to pre-existing, frequently subclinical, chronic kidney disease (CKD), or may be the cause of CKD [1,2,3]. Regardless of the cause, kidney injury is an important obstetric complication associated with significant maternal and foetal morbidity and mortality [4]. Pregnancy and AKI pose a challenging clinical scenario, where the effects on maternal and foetal outcomes need to be monitored closely. Women with kidney injury should be considered high-risk and are ideally managed by a multidisciplinary team involving nephrology and obstetrics staff [5].

Understanding the physiologic changes in pregnancy in contrast to pregnancy-mediated alterations of renal function that occur in women with renal disease forms the basis of the best care of pregnancy. It is essential that the team providing care are familiar with systemic and renal physiological adaptive mechanisms, laboratory parameters in pregnancy, and are knowledgeable on the mainstay therapeutic measures to be initiated in the context of suspected or actual pregnancy-related acute kidney injury (PR-AKI). The changing landscape of the epidemiological and clinical characteristics of the pregnant population has impacted the incidence, etiology, foetal, and maternal outcomes of PR-AKI and calls for changes in approach to management and clinical practice in order to achieve the best outcomes.

Recent published literature demonstrates the increased awareness of kidney injury in pregnancy and the management of PR-AKI, with focus on the important relationship between pre-eclampsia, pregnancy-induced hypertensive disorders, thrombotic angiopathies, and AKI [1,6,7,8,9]. 

This review provides an overview of the changing epidemiological and clinical landscape of acute kidney injury in pregnancy, followed by specific discussions on recent recommendations in the approach to the management of this cohort of women.

## 2. Physiological Changes and Kidney Adaptation to Pregnancy

**Figure 1:**
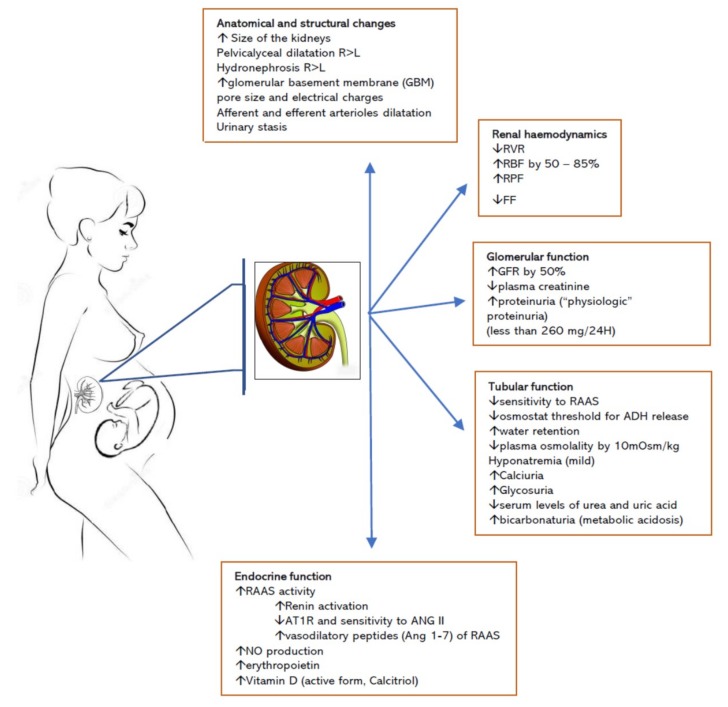
Renal adaptation to pregnancy. Abbreviations: GFR, glomerular filtration rate; RVR, renal vascular resistance; RBF, renal blood flow; RPF, renal plasma flow; FF, filtration fraction; RAAS, renin-angiotensin system; AT1R, angiotension type 1 receptors.

The kidneys are the main players in the physiological adaptation to pregnancy, all aspects of kidney function undergo dynamic changes that contribute to the specific environmental milieu of the pregnant woman and the foetus. Understanding the structural and functional alterations of the renal system that occur throughout pregnancy is essential in the evaluation and management of pregnant women with or without renal disease (Figure 1).

***Anatomical changes:*** The kidneys increase in size during pregnancy and return to the pre-pregnancy dimensions within 6 months after delivery. The overall volume of the kidneys increases by up to 30%. The bipolar diameter increases by approximately 1.0 to 1.5 cm. There is also an increase in the size of the kidney pelvis, of 2 cm on the right and slightly less, of about 1.5 cm, on the left kidney [10]. The growth is attributable to the increase in the vascular and interstitial volume as well as to pelvicalyceal dilatation [11], while the number of nephrons does not change [12]. A degree of hydronephrosis is common during pregnancy, affecting more than 60% of women, with a maximum incidence at 28 weeks gestation [13]. The hydronephrosis is predominant on the right side, and is due to a combination of the effects of progesterone and mechanical compression elicited by the gravid uterus at the pelvic brim [14] (Figure 1).

***Adaptation of renal haemodynamics during pregnancy***: The pregnancy is a state characterized by volume expansion and vasodilation [15]. In the non-pregnant state, renal blood flow represents 20%–25% from the cardiac output although kidneys weight less than 1% of the body mass. In pregnancy, the cardiac output increases by 40%–50% by week 26 through a combination of increased heart rate (10%–20%) and stroke volume (20%) [16]. At the same time, there is a reduction in systemic blood pressure due to significant decrease in systemic vascular resistance. With these changes, there is a fall in renal vascular resistance and a 70%–80% increase in renal blood flow, which picks at the end of the second trimester before decreasing in the third trimester. Consequently, effective renal plasma flow increases by 60%–80% by mid-pregnancy, whereas the increase in glomerular filtration rate (GFR) is slightly less, about 40%–50% by the end of second trimester [16]. As such, the filtration fraction (FF), the ratio between GFR and renal plasma flow, is altered, decreasing in the first half of pregnancy only to increase in the late gestation. The haemodynamic variables recover to non-pregnant state by 4 to 6 weeks after delivery (Figure 1 and Table 1). 

***Adaptation of glomerular function during pregnancy:*** In humans, the GFR increases as early as 6 weeks after conception and is usually sustained until the end of gestation. The rise in GFR precedes plasma volume expansion and the increase of the cardiac output [19]. The overall increase in GFR in pregnancy is 40% to 60%. However, data reported show considerable individual variation [1] and reference curves of kidney function in pregnancy are still to be defined [2,3]. The mechanisms leading to the GFR changes are complex and not fully understood [17], and are related to changes in capillary hydrostatic pressure, oncotic pressure, and glomerular flow [17]. The increase in GFR leads to increase in urine volume in 24 h, delivery of higher volume of solutes and electrolytes to the lumen of the renal tubules, and an increase in the renal excretion of amino acids, glucose, proteins, electrolytes, with consequent reduction in their serum levels (Table 2). 

In the general population, calculated GFR is used as a proxy for assessment of renal function. Traditional methods used to measure actual GFR such as inulin or iohexol clearance (gold standard), 24 h creatinine clearance, are cumbersome and not practical or available for routine practice. Thus, serum creatinine is used to estimate GFR in daily patient care, facilitating the diagnosis of kidney injury and grading the severity of the disease. However, the calculations for GFR estimations from serum creatinine concentrations are not validated in pregnancy and underestimate the GFR to varying degrees depending on the stage of pregnancy [24]. It is also worth noting that normal creatinine ranges in isolation are not useful for defining renal dysfunction and often PR-AKI can only be defined by using intra-variability of creatinine levels in the individual pregnant women.

Though there have been several studies that aimed at defining the reference values for serum creatinine, none are considered accurate in pregnancy [23,25,26]. A recent systematic review found that, using a nonpregnant reference interval of 45–90 mmol/L (0.51–1.02 mg/dL), a serum creatinine higher than 77 mmol/L (0.87 mg/dL) should be considered pathological, outside the normal range for pregnancy [23] (Table 1).

***Adaptation of tubular function during pregnancy:*** The alterations of tubular function during pregnancy manifest as an increase in excretion of solutes and electrolytes. Mild glycosuria is present in about 10% of women with normal blood glucose [27]. In normal pregnancy there is a slight increase in total urinary protein and albumin excretion, which varies from less than 100 mg up to 200–300 mg protein per 24 h [28]. Proteinuria higher than 300 mg per 24 h however, is always considered abnormal, although protein excretion does not always correlate with the severity of the renal disease [28]. Normal pregnancy is also associated with bicarbonaturia, calciuria, and metabolic acidosis that offsets the respiratory alkalosis due to changes in ventilation of pregnant women [11,18]. The osmostat is reset in pregnancy to lower values of osmolality. Plasma osmolality drops from 290 mOsm/kg to 280 mOsm/kg, whereas there is a net retention of electrolytes in pregnancy, of 900–950 mEq/L for sodium and 300–350 mEq/L for potassium [20,29,30] (Table 1).

## 3. The Changing Epidemiological Landscape of Acute Kidney Injury in Pregnancy

Acute kidney injury (AKI) is defined as an acute, reversible increase in serum blood urea nitrogen and creatinine and/or oliguria [31]. Several AKI classification systems (Risk, Injury, Failure, loss (RIFLE), Acute Kidney Injury Network (AKIN), and Kidney Disease: Improving Global Outcomes (KDIGO) have been developed to overcome the shortcomings of this vague definition and enable a consensus between researchers and clinicians [32,33,34,35,36] (Table 3 and Table 4). Review of the classifications and comparisons of the three systems discriminative ability for the prediction of patients’ outcomes and hospital mortality is provided elsewhere [37,38,39]. Overall, it was found that all three systems have good performance in outcome prediction, with KDIGO giving higher estimate and having relatively higher discriminatory power than the other two classifications. However, the varying methods of estimating AKI, as well as varying demographics of the studies could also have contributed to variation in the outcomes. 

RIFLE criteria were developed by the Acute Dialysis Quality Initiative (ADQI) in 2002 [36]. AKIN criteria were developed by the Acute Kidney Injury Network in 2004 [33]. KDIGO criteria were developed as clinical practice guidelines for AKI by the Kidney Disease Improving Global Outcomes in 2012 and were built off of the RIFLE and AKIN criteria [36].

Although the true incidence is possibly underestimated, it is appreciated that AKI affects 10%–20% of hospitalized adults and up to 60% of the critically ill patients [34,40]. AKI is associated in non-pregnant patients with an increased risk of CKD and further end-organ complications [41], and remains one of the major pathological factors in clinical practice worldwide, with rates of mortality as high as 20%–40% [31,42,43].

PR-AKI is a term defining AKI occurring during pregnancy, labour and delivery, and/or the postpartum period. The incidence of PR-AKI has sharply decreased over the decades. In the 1960s and 1970s, PR-AKI was reported as having an incidence between 6% and 50% of pregnancies [41,44] 15% in the 1980s [44] and 1.5%–1.8% in 2010 [9,42,45]. PR-AKI is a heterogeneous disease entity with a multitude of underlying aetiologies that poses serious problems to pregnancy, due to increased risk of maternal and foetal complications and high mortality rate [1,45]. The rates of maternal mortality and foetal loss in patients with PR-AKI have been reported rising from 6% to 30%–60% by some studies [2,45,46].

PR-AKI is clearly more common in the developing nations, with a reported incidence of 4%–26% versus an incidence of 1.0%–2.8% in developed countries [47,48]. Poverty, multiparity, overcrowding, poor education, lack of proper healthcare facilities and resources, lack of awareness of the condition, deficiencies in obstetric and medical care, delayed referral processes, and transport issues are among the common socioeconomic and health care factors contributing to PR-AKI in low-income countries [49]. Considerable improvements in obstetric, prenatal, and medical care, as well as a decline in the rate of septic illegal abortion, have decreased the incidence of PR-AKI worldwide during the past 50 years [50]. The incidence was reduced from 20% to 40% in the 1960s to less than 10% in more recent years [45].

Changing trends in the epidemiology of PR-AKI have been observed over the past years with global variations in the predominant causes of PR-AKI. Though the incidence remains higher in developing countries, this appears to be decreasing, while PR-AKI appears to be on the rise in developed countries. The changes in the developed nations may be attributable, at least in part, to the changing demographics of the pregnant population and the combination of the women and the clinicians extending the boundaries of “acceptable” pre-pregnancy states. The changing demographics of the pregnant population include older women with more pre-existing comorbidities such as obesity, diabetes, chronic hypertension, and chronic kidney disease [51]. Increased rates of assisted reproduction, particularly ovulation induction and egg donation are becoming potential causes of PR-AKI in developed countries due to complications associated with these procedures and subsequent renal damage [52,53,54,55].

An Italian study from 1996 reported a decrease in the incidence of AKI in pregnancy from 1:3000 to 1:18,000 births from the 1960s to 1990s [56]. More recent reports revealed an increasing incidence of pregnancy-related AKI of 61%, from 1.66 to 2.68 per 10,000 deliveries between 2003 to 2010 in Canada and from 2.4 to 6.3 per 10,000 deliveries between 1999 to 2011 in the United States [57,58]. This upward trend was partly attributed to increased surveillance with prompt diagnosis and ascertainment bias [59]. Fortunately, the majority of cases were minor and transient and the increase was restricted to women with hypertensive disorders, particularly women with pre-eclampsia, whereas the increase among women without hypertensive disorders was rather marginal (12%) [57].

Improved perinatal surveillance in developing countries is reflected in decreasing rates of women who develop PR-AKI during puerperium. A retrospective Chinese study reported an incidence of PR-AKI to range from 0.2% to 1.8% [60], while India has shown an even more sharp decline in the proportion of PR-AKI among hospitalized patients, from 15% in the 1980s to 1.5% in the 2010s [61,62]. A recent prospective study from Morocco reported an incidence of PR-AKI of 6.6 per 1000 deliveries [63]. Although these studies report a decrease in the occurrence of PR-AKI in developing countries, maternal morbidity, and mortality associated with PR-AKI remains unacceptably high in both developed and developing countries. In the United States, the increase in severe PR-AKI requiring dialysis was from 0.27 to 0.36 per 10,000 deliveries and the increase in maternal mortality associated with PR-AKI was from 0.13 to 0.23 per 10,000 deliveries [58]. In Canada, the incidence of severe PR-AKI requiring dialysis was lower, 1 in 10,000 pregnancies, but maternal mortality rates were higher, of 4.3%, than 0.01% in the general population [64]. The incidence of severe PR-AKI requiring dialysis, as a proxy measure for maternal morbidity and maternal mortality rates, was reported much higher in the developing countries. In a Moroccan study, haemodialysis was necessary in 16.2% of cases [63]. Thirty percent of women with severe PR-AKI required dialysis in an Indian cohort [62], whereas the rate of dialysis was only 6% in Chinese women [60].

The changing pattern of the etiology of PR-AKI is worth highlighting. An evolution in the characteristics of kidney injury in pregnancy is apparent, with a decline in AKI in early pregnancy due to abortion and puerperal sepsis-related causes but an increase in frequency of hypertensive disorders, thrombotic microangiopathies, and postpartum—associated AKI. These changes reflect not only advances in obstetric care, but also possible modifications in underlying maternal risk factors such as advanced maternal age, increased use of reproductive technologies, and increased incidence of non-communicable diseases including hypertension, diabetes, obesity, and chronic kidney disease [9,65].

## 4. Challenges in Diagnosis of PR-AKI

To date, there is no standardized definition of PR-AKI in literature [66]. The international consensus classifications mentioned above and illustrated in Table 3 and Table 4 are used for defining and staging AKI in non-pregnant patients and cannot be extrapolated to pregnancy. The rapid deterioration of kidney function that characterizes AKI has been variably defined in pregnancy, either as an increase in serum creatinine over 80 mg/dL, doubling in serum creatinine levels, oliguria of <400 mL/24 h or the need of dialysis [23,66,67]. The lack of consensus in PR-AKI definitions arises from the physiological variability in the cardiovascular haemodynamics and renal function changes in the pregnant population. Whereas in non-pregnant patients, glomerular filtration rate (GFR) estimated from serum creatinine levels is routinely used to evaluate kidney function, in pregnancy, the use of this renal variable is less accurate, when GFR consistently underestimate renal function. Furthermore, in comparison to the non-pregnant reference interval of 0.51–1.02 mg/dL, the serum creatinine levels are overall lower in pregnancy, with values in the third trimester as low as 0.4–0.8 mg/dL, due to a combination of physiological alterations that include blood volume expansion, hyperfiltration, and decrease in oncotic pressure [23,65]. In addition, the reference values of serum creatinine in pregnancy are still under debate. A recent systematic review and meta-analysis found that serum creatinine levels higher than 77 μmol/L (0.87 mg/dL) should be considered outside the normal range for pregnancy [23]. A more recent Canadian study, aiming to define gestational age-specific estimates of renal function, reported the ranges of serum creatinine in normal term pregnancy between 53–70 μmol/L, with an average of 61 μmol/L [26]. Therefore, acute increase in levels of creatinine during pregnancy above these levels, in an otherwise healthy woman, should prompt investigations into the cause of such occurrence. 

**Figure 2:**
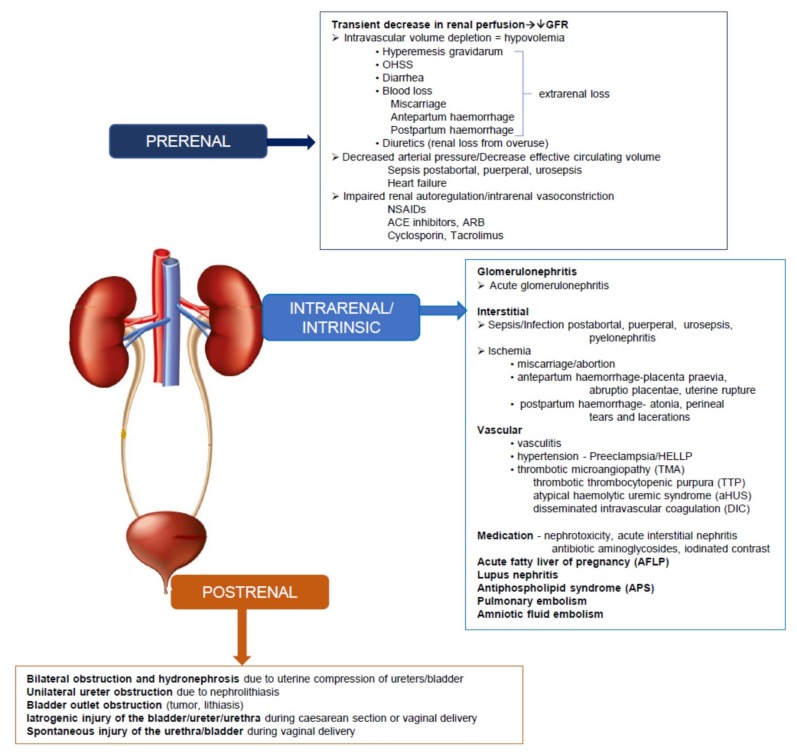
Common causes of pregnancy-related (PR)-AKI classified by a physiopathological mechanism of kidney injury.

***PR-AKI Etiology*** is multifactorial and complex, as kidney injury may occur at any time during pregnancy or in the postpartum period. A higher incidence of PR-AKI was observed towards the end of the third trimester and around the time of delivery. Whereas in developed countries, pregnancy-induced hypertensive disorders are the leading etiology, in developing countries sepsis and haemorrhage are main contributors [61,68]. A study from India reported that PR-AKI represented 3% to 7% of the cases of AKI in the Indian sub-continent, 60% of which occurred in the postpartum period and 32% in the third trimester [61]. Sepsis (59%), pre-eclampsia, and eclampsia (56%) were the leading causes of PR-AKI from this report, while sepsis was the leading cause of maternal mortality. Maternal and foetal mortality were reported as 20% and 22%, respectively. From the PR-AKI cases, 36% progressed to renal failure, among which 30% required renal replacement therapy (RRT) [61] (Figure 2).

Diseases or conditions that are specific and unique to pregnancy, such as pregnancy-induced hypertensive complications, intrauterine death, and haemorrhage play a predominant primary role in the genesis of PR-AKI. A second etiological category is comprised of diseases or conditions that are not specific to pregnancy but happen to coincide with pregnancy. In the latter, pregnancy acts as a trigger for PR-AKI, mediated by genetic, immunologic, or constitutional factors. An alternative classification of PR-AKI includes as criteria the time when the complication occurs during pregnancy: (i) early, first trimester (hyperemesis gravidarum, miscarriage); (ii) late, end of second and third trimester (placental abruption, and microangiopathies); (iii) postpartum (uterine atonia, vaginal and perineal tears); and (iv) anytime (various causes of hypovolemia, sepsis, glomerular, interstitial, or tubular kidney damage [1,9] (Table 5).

The etiology of PR-AKI may also be categorized by a pathological mechanism based on the location of the pathology, similar to non-pregnant populations, in pre-renal, renal, and post-renal pathologies. An in-depth discussion of PR-AKI based on the causes and the pathophysiological mechanism is beyond the scope of this manuscript. We guide the reader to recently published reviews for an extensive account of the PR-AKI aetiologies [1,6,8,9] (Figure 2).

In summary, clinicians who provide care to pregnant patients should be aware that an acute reduction in renal function (i.e., a sudden increase in serum creatinine levels) in pregnancy may occur from any of the causes associated with the non-gravid state as well as from a number of disorders specific to pregnancy. Elucidating the etiology of AKI is of the utmost importance in pregnancy because once the diagnosis is made the cure can be promptly initiated. The longer the duration of AKI, the higher the reduction of nephron mass, and the higher the chances of developing CKD later in life [69].

## 5. Principle of Management of PR-AKI

***The diagnosis of PR-AKI*** is difficult and the management complex as there are two patients to consider, the mother and the foetus. Furthermore, the diagnosis is challenged by the differentials between pregnancy-associated specific diseases and other causes of AKI that might affect any women of reproductive age. Therefore, a multidisciplinary team of specialists is warranted for providing care for these patients, which should include maternal–foetal and obstetric medicine, nephrology, neonatology, and where necessary, anaesthetics and critical care. Midwives and nurse specialists are also key components to the complex care needs these patients require.

***General principle of management.*** Once the diagnosis of PR-AKI is confirmed, the management should be promptly implemented in several key steps, with the early involvement of nephrologists [9,66]. The leading principles of PR-AKI treatment are stabilization of the patient, treatment of pathology where possible, and prevention of further kidney damage. Maintaining supportive care and foetal wellbeing monitoring complete the main steps in PR-AKI management. Although maternal health takes priority, foetal wellbeing should also be monitored and optimized. Timely initiation of renal replacement therapy (RRT) if required and, specific to pregnancy, prompt delivery of the foetus if necessary, complete the arsenal of PR-AKI management [66]. It is also important that the team is aware of the potential impact on the emotional and mental wellbeing of pregnant women with PR-AKI. Avenues and pathways may be required for supportive therapy such as psychological support and counselling in the short- and medium-term. Furthermore, there is considerable acceptable variation in inter-patient and inter-unit approaches to management of PR-AKI. It is, however, essential that the underlying cause is taken into account and basic principles are followed in identifying pathological as opposed to physiological adaptions, particularly to enable the appropriate approach to fluid and electrolyte management.

The PR-AKI patient requires respiratory, circulatory, and haemodynamic assessment that together with haematologic and biochemical laboratory investigations direct the therapy to restore the haemodynamic stability. Close clinical monitoring of haemodynamic status, urine output, and respiratory function is essential to inform volume resuscitation needs in order to maintain adequate renal perfusion, prevent further deterioration, and reverse pre-ischemic renal changes. Volume resuscitation involves oral intake of fluids (where possible) and parenteral administration of crystalloid and colloid solutions. Ideally, an adequate fluid balance should be monitored and maintained with isotonic solutions such as normal saline or Hartmann’s solution rather than hypertonic or hyperoncotic solutions [70]. Blood products should only be administered if indicated by the clinical status of the patient and laboratory tests, in accordance with local guidelines. While in most cases these patients are cared for in high-dependency obstetric units, invasive haemodynamic monitoring in intensive critical care units is warranted for more complicated cases [70,71].

The identification and treatment of the underlying cause of PR-AKI may require specific tests to complete the panel of investigations. The most common pathophysiological mechanism of PR-AKI is, however, reduced renal perfusion. Haemorrhage or other pregnancy-related specific causes such as hypertensive complications are most of the time readily apparent, facilitating the diagnosis and offering the opportunity of prompt intervention by a series of measures that include, but are not limited to, arresting the ongoing bleeding, instituting more aggressive antihypertensive therapy, or even delivery of the foetus if safe to do so. Renal ultrasound should be considered in most patients with AKI to identify obstruction. Specific appropriate treatments should be administered according to the pre-existing co-morbidities. Treatment of underlying diseases may not only contribute to re-establish kidney function but will also act to resolve recurrence or prevent further deterioration of pre-existing diseases that complicate the pregnancy [9,70].

The measures to prevent the progression of kidney damage partly overlap with the steps described above. Maintaining adequate perfusion of the kidney may require, in addition to fluid resuscitation, administration of vasoactive drugs and diuretics. In cases of post-renal PR-AKI caused most frequently by obstruction of the urinary tract, either by the gravid uterus or by urinary stones, placement of ureteral stents, percutaneous nephrostomy, or delivery of the foetus if indicated (i.e., patient near term) may resolve the kidney injury. Of note, caution is required as not all the urological procedures used in non-pregnant patients have yet been validated for safe use in pregnancy [72,73,74], and close liaison with urology and radiology colleagues is advised.

***The complications of PR-AKI*** are similar to those of AKI in non-pregnant patients and include volume overload, electrolyte, acid-base, and nutritional disturbances, anaemia, and an increased risk of infection. These complications are maybe difficult to differentiate from the underlying causes of kidney injury. PR-AKI complications can be treated the same way as in non-pregnant patients. 

***Anaemia*** may develop rapidly and is frequent in patients with acute kidney failure. The etiology of anaemia in AKI is multifactorial, contributors include low iron and vitamin stores, reduced erythropoiesis, reduced red blood cells survival time, bleeding diatheses due to thrombocytopenia, platelet dysfunction, in some cases coagulopathy, haemolysis, haemodilution, and frequent phlebotomy for blood tests. The treatment of acute anaemia in AKI is with red blood cell transfusions; iron and folic acid supplements may be added to sustain erythropoiesis. Routine administration of exogeneous erythropoiesis-stimulating agents may not benefit survival or other long-term clinical outcomes and are not routinely recommended in pregnancy. These agents may be considered, however, in patients with chronic anaemia [75,76], although higher doses might be required to obtain a therapeutic effect [7]. Anaemia does not increase the risk of progression from early AKI to more severe AKI [76].

***Volume overload*** is a consequence of avid resuscitation in hypotensive patients with oliguric or anuric AKI. In clinical practice, volume overload may result from intravenous fluid infusions administered to re-establish diuresis, infusions of medications such as antibiotics, vasopressors, nutritional support, or administration of excessive volume of maintenance fluids, less commonly as a result of excess oral intake. Increased central venous pressure, peripheral oedema, pulmonary congestion and oedema, and accumulation of fluid in third spaces (peritoneum, pleura) point to the diagnosis. Limiting fluid administration is usually enough to optimize balance. In some situations, a trial of diuretics administered intravenously as a bolus or continuous infusion (i.e., loop-acting diuretics such as furosemide, alone or in combination with thiazide or thiazide-like diuretics) may be indicated obtain the fluid balance in same patients [77]. It is important to underline here that diuretics should not be used to prevent or treat AKI in absence of volume overload as they do not improve the renal outcomes, morbidity, or mortality [78,79]. RRT should be considered in patients who do not respond to diuretic therapy or in those who develop progressive volume overload despite fluid restriction and diuretic therapy or remain anuric and develop azotaemia [77]. Fluid management in pre-eclampsia poses particular challenges due to heightened risk of volume overload and pulmonary oedema in these women. The management algorithm in these cases should set limits to the total volume administered with a view to input matching urine output and insensible fluid loss. A fluid balance chart is essential in these cases [80].

***Electrolyte disturbances***, hyperkalaemia is the most commonly affected in AKI and requires prompt intervention. The etiology of hyperkalaemia is multifactorial, with impaired renal excretion of potassium and efflux of potassium from the intracellular compartment (the result of coexistent acidosis), hyperglycaemia, and hyperosmolarity, being the most common causes. Due to its potential impact on cardiac arrhythmia/arrests, hyperkalaemia should be promptly corrected with intravenous infusion of insulin and glucose (to prevent hypoglycaemia in patients who are not hyperglycaemic) and potassium-binding resins, according to the unit guidelines and involving the nephrologists. Loop diuretics are used in selected cases to enhance potassium excretion. Intravenous calcium and catecholamines complete the treatment [81,82]. In some cases, there may be a need to restrict dietary potassium intake. Caution should be exercised in the use of diuretics, as commonest causes of PR-AKI cause hypovolemia.

Other electrolyte disturbances in AKI involve sodium and calcium. Hyponatremia is common, whereas hypernatremia is rarely seen. Hyponatremia is usually mild and is resolved by free-water restriction. Hypocalcaemia is typically asymptomatic and does not require specific treatment. If symptomatic, it is treated with intravenous calcium, with attentive monitoring of serum phosphate, as hyperphosphatemia may also be present in AKI and may require treatment with phosphate binders, especially in the context of acute on chronic kidney disease [83].

***Metabolic acidosis*** is the most common acid-base disturbance associated with AKI due to impaired excretion of acid, although the underlying cause of AKI may exacerbate the acidosis and increase the anion-gap. Treatment of the underlying cause and administration of bicarbonate solution corrects the acidosis in most cases [84]. Although the administration of bicarbonate solutions has become a common practice to correct acidosis in AKI, the evidence from a recent systematic review could not support or refute the use of sodium bicarbonate in patients with AKI [84]. Of note, correction of metabolic acidosis in pregnant patient should take into consideration the physiological status of respiratory alkalosis in pregnant patients. However, there are no clear recommendations on the use of bicarbonate in pregnancy, and its use in pregnant patients should be decided in consultation with a nephrologist and obstetric medicine specialist. 

***Nutrition*** support needs to be given consideration as AKI is considered a hypercatabolic state and anorexia, nausea, and vomiting are frequently present. In situations where enteral feeding cannot be achieved, parenteral nutrition should be considered, aiming to provide the necessary caloric and protein intake [85,86]. 

***Indications for RRT/dialysis*** in PR-AKI patients are similar to non-pregnant patients and are represented by volume overload non-responsive to diuretic therapy, hyperkalaemia and metabolic acidosis refractive to medical management, and symptomatic uraemia (manifested as pericarditis, encephalopathy, or neuropathy). Any modality of dialysis can be used in pregnancy, with improved pregnancy and foetal outcomes reported for both, haemodialysis and peritoneal dialysis [87,88]. There may be, however, limiting factors to peritoneal dialysis in the last trimester due to capacity issues in the abdomen with the enlarging gravid uterus. Although there are no randomized trials to show benefits for a specific technique, haemodialysis is the most common method used in PR-AKI [87,88]. RRT in PR-AKI has several particularities: (i) it is often prescribed for short-term; (ii) an increase in the duration and frequency of dialysis sessions is recommended, targeting a serum urea between 45–60 mg/dL; and (iii) the aim is to avoid excessive fluid shifts and hypotension which can affect the fetoplacental circulation and influence foetal wellbeing [88]. 

***Foetal monitoring***. Along with the management of the mother, monitoring of foetal growth and wellbeing is an integral part of the care plan in PR-AKI. This should be initiated in close consultation with neonatologists and foetal medicine specialists. Foetal well-being and neonatal outcomes are closely linked to maternal status. Prompt treatment of volume depletion and hypotension in mother ensures adequate blood flow to the uterus and fetoplacental unit. The degree of foetal monitoring is dictated by the gestational age and extent of foetal compromise. Foetal heart rate monitoring should be instituted daily for the viable pregnancies from 23–24 weeks gestation. From 26 weeks onward, foetal heart rate can be monitored via cardiotocography, frequency of which is dependent on the foetal status. The frequency of ultrasound and biophysical profile may be established depending on the clinical situation. If delivery is indicated between 24- and 32-week gestation, magnesium sulphate should be considered, and if indicated between 24- and 34-week gestation, corticosteroids should be administered to reduce neonatal morbidity and mortality [8,9,53].

## 6. Clinical Presentations of PR-AKI

The diagnosis of PR-AKI follows a bimodal distribution, with two peaks of increased incidence: one in the first trimester, predominantly caused by hyperemesis and septic abortion, and the other in the third trimester and postpartum, caused by pregnancy complications and postpartum haemorrhage [7]. This section highlights some of the specific causes of PR-AKI [6], discussing in more detail bleeding and sepsis throughout pregnancy, hypertensive disorders, and haematologic immune conditions [6,89].

### 6.1. Bleeding and Hypovolemia

Less than 5% of PR-AKI are caused by volume depletion in the first trimester. In developed countries, hypovolemia caused by haemorrhage from a miscarriage in the first trimester is less commonly a cause of pre-renal azotaemia. Excessive blood loss is usually from second-trimester miscarriage and antepartum haemorrhage from placenta praevia or placental abruption [6,8,89,90]. Some reports estimate that most PR-AKI occurs in the postpartum period (68%) and results from the peripartum blood loss with volume depletion and pre-renal insults. Besides uterine atony, which is the most common, retained placenta, genital tract lacerations, coagulopathy, placenta accreta, uterine rupture, uterine inversion, and coagulopathy from an amniotic fluid embolus are among the causes of bleeding after delivery of the foetus. Frequent underestimation of blood loss at delivery leads to difficulty in prompt identification of PR-AKI in such cases [32,41,42,57].

These require resuscitation with blood transfusions and clotting factors and may require additional surgery if indicated (see also Figure 2).

Besides haemorrhage, intravascular volume depletion in hyperemesis, ovarian hyperstimulation syndrome (OHSS), are common causes of pre-renal azotaemia in early pregnancy. 

Persistent vomiting associated with metabolic alkalosis in hyperemesis gravidarum benefits from antiemetic therapy and volume replacement with crystalloid solution and often potassium supplementation [45,91]. Severe refractory to medical treatment or untreated cases may lead to severe dehydration, hypovolemia, and pre-renal AKI whose treatment may require haemodialysis [92,93].

The incidence of OHSS is increasing with increasing rates of IVF, and is expected to rise as more women with chronic comorbid conditions that can be exacerbated by the pregnancy aim to procreate using gestational carriers [94]. Severe OHSS is frequently complicated by AKI, the mechanisms of which are complex and include intravascular volume depletion, humorally-mediated capillary leak leading to kidney oedema and intra-abdominal hypertension or compartment syndrome, and obstructive uropathy due to ovarian enlargement [94]. A role for VEGF in altering vascular permeability and facilitate the pathophysiological changes in OHSS, including AKI has recently emerged [95]. Management strategy involves careful rehydration to re-establish fluid and electrolyte balance, antiemetics, steroids, and low molecular dose heparin to prevent thrombosis. The role of thoracentesis, paracentesis, and haemodialysis is assessed on an individual basis [96]. 

### 6.2. Infection and Sepsis

Some studies report infection and sepsis among the most common causes of AKI in pregnancy, which may include pyelonephritis, intrapartum, postnatal or post-abortion sepsis, or abscess [50,97]. Bleeding and infection, most frequently with Gram-negatives such as *Escherichia coli* and *Clostridium* spp., following a septic abortion or uterine abscess can lead to severe tubular necrosis that requires antibiotics, fluid resuscitation, pressors for hypotension, and, eventually, dialysis until renal function recovers [45]. An updated detailed account of the pathophysiology and management of acute tubular necrosis is provided in a recent review by Hanif and Ramphul [98].

Acute pyelonephritis occurs in approximately 1%–2% of pregnancies, more frequently in the second trimester. *E. coli* is the predominant causative agent for acute pyelonephritis (70%). Other pathogens involved are *Klebsiella* and *Enterobacter* (3%), *Proteus* species (2%), *Staphylococcus*, and (group B) *Streptococcus* (10%) [66,99]. In contrast with the uncomplicated urinary tract infections such as asymptomatic bacteriuria and cystitis, which are resolved with oral antibiotics, a diagnosis of pyelonephritis requires hospital admission, parenteral antibiotics, and fluids, due to high-risk of these patients to develop profound septic syndrome with a reduction in the GFR [66]. Thus, the treatment should be promptly initiated, with hydration and antibiotics selected based on microbiology input on bacterial species cultured and their sensitivity. Implementation of aggressive management principles as described above has led to a reduction of pyelonephritis-induced PR-AKI from 20% to 2% [100] and had been shown to reverse the changes in the GFR. It is important to note the risk of pulmonary oedema and acute respiratory distress syndrome in women who develop sepsis, particularly those with Gram-negative urosepsis, result of alteration of the alveolar-capillary membrane permeability caused by endotoxins [101]. These patients frequently require transfer to critical care units for supportive care, intubation, and mechanical ventilation. Diuretic therapy, if indicated, should be used with extreme caution [9]. The reviews by Rao and Jim [9], and Jim and Garovic [66] offer a comprehensive discussion on pyelonephritis in pregnancy and the antibiotic therapy selection. It is important to note that the antimicrobial therapy is effective only in eliminating bacteria, but not in treating issues with reflux nephropathy which could lead to scar formation and recurrent pyelonephritis episodes, which may lead to long-term complications due to post-pyelonephritic renal scarring [102].

Hypovolemic status from bleeding and dehydration leads to systemic hypotension and renal hypoperfusion that alone or combined with vasoconstriction, release of inflammatory cytokines and reactive oxygen species in sepsis cause acute tubular necrosis, the most common causal pathway of AKI [98]. Urinalysis with granular casts and an elevated fractional excretion of sodium help to confirm a suspected diagnosis of acute tubular necrosis in clinical context. Beside fluid resuscitation and antibiotics, the treatment includes pressors to correct the hypotension and, if necessary, dialysis until renal function recovers [98]. Acute tubular necrosis as a cause of PR-AKI in late pregnancy is less common, usually associated with pre-eclampsia, haemolysis, elevated liver enzymes, and low platelet count (HELLP) syndrome or uterine haemorrhage in abruptio placentae [103]. 

Septic abortion and obstetric emergencies such as abruption placentae, especially if they occur in older, multiparous women, or in multiple pregnancies may lead to renal cortical necrosis, a rarer cause of severe PR-AKI [104]. Prolonged intrauterine foetal death, eclampsia, and postpartum haemorrhage are additional pregnancy-related risk factors [105]. Renal cortical necrosis accounts for more than 20% of acute renal failure during the third trimester of pregnancy [8,106]. The aim of therapy in renal cortical necrosis is to restore hemodynamic stability, treat the underlying cause, and promptly institute dialysis in order to recover renal function [107]. Association of primary disseminated intravascular coagulation as a primary initiating event hinders recovery in these cases, cortical necrosis requiring thereafter months for renal functional recovery, which is usually incomplete [108].

### 6.3. Hypertensive Disorders of Pregnancy

Pre-eclampsia, eclampsia, HELLP syndrome, and acute fatty liver of pregnancy are among the most important causes of kidney injury in late pregnancy, predominantly third trimester, although they also occur in postpartum. Clinical manifestations, pathophysiological mechanisms, specific investigations, and management principles have been thoroughly reviewed [1,9,53,66]. Thus, we refer the reader to the abundant recent literature on the subject. Nevertheless, pre-eclampsia, acute fatty liver of pregnancy, HELLP syndrome, and the thrombotic microangiopathies (thrombotic thrombocytopenic purpura and atypical haemolytic uremic syndrome) exhibit overlapping features which challenge the diagnosis. 

### 6.4. Haematological/Immune Conditions

Microangiopathic haemolytic anaemia, the hallmark of thrombotic microangiopathy, is a group of conditions that include haemolytic uremic syndrome (HUS) and thrombotic thrombocytopenic purpura (TTP). Pregnancy-associated thrombotic microangiopathies are rare, affecting 1 per 25,000 pregnancies [109]. The two conditions, HUS and TTP have similar clinical manifestations, that frequently overlap and may hinder the distinction in daily practice. The difficulty arises from having the correct diagnosis as the two conditions have completely different treatment [110,111]. TTP is characterized by severe thrombocytopenia and non-specific neurologic symptoms such as seizures, aphasia, ataxia, disorientation, and headaches, and, most usually, mild renal failure (serum creatinine < 1.4 mg/dL) [111]. Deficiency of ADAMTS-13, a von Willebrand factor–cleaving protease, is responsible for most cases of TTP. TTP has a maximum incidence during the last week of pregnancy and is treated with plasma exchange or fresh frozen plasma infusions for clearance of autoantibodies and restoration of enzymatic activity [9,112]. In contrast, HUS is characterised by a more pronounced renal involvement (AKI with high serum creatinine, usually >2.3 mg/dL), exhibiting rarely neurological symptoms [9,111]. HUS develops more commonly in postpartum and the mechanism involves an excessive activation of the alternative complement pathways with genetic mutations in the complement regulatory proteins, such as complement factor H, I, C3, and membrane cofactor protein [113]. Similar to TTP, plasma exchange therapy was considered the “gold-standard” for the treatment of HUS in the past. Plasma exchange was used in HUS to replace the non-functioning complement regulators and remove the autoantibodies. It has been shown to induce remission in 50% to 80% of cases but was not a definitive treatment. Some of the patients with atypical HUS have specific genetic anomalies that respond better to kidney transplant [114,115]. The advent of monoclonal antibodies therapies has revolutionized the management of HUS over the past several years. The new therapeutic agent is Eculizumab, a monoclonal antibody which targets the C5 fraction of the complement system, preventing the cleavage of this complement fraction, and generation of the membrane attack complex [116]. Eculizumab use was shown to be safe in pregnancy and can achieve clinical remission or even cause reversal of HUS [9,117]. The treatment with Eculizumab in pregnancy has not been yet standardized in regard to the duration of treatment or its use as prophylactic therapy to prevent recurrence in subsequent pregnancies [9].

## 7. The Risk of CKD after AKI

In addition to the short-term risks, there is accumulating evidence on the long-term adverse consequences and the risk of progression of AKI [118,119,120]. Retrospective analyses of historical administrative datasets have shown the association between AKI and increased risk of CKD, cardiovascular diseases, and mortality. These findings are supported by recent prospective studies, that by reducing the ascertainment and confounding bias were able to confirm the relationship between AKI and risk of CKD. For instance, a prospective cohort of an unselected population attending a general hospital found a deterioration in renal function at 3 years in 30% of patient diagnosed with AKI. Of note, this association was present even in individuals who sustained stage 1 AKI, thus, demonstrating the relevance of potential disease progression even for less severe renal injury [119]. It is possible that more patients are affected in longer-term follow-up [119]. A systematic review showed that the risk for CKD increases progressively with the severity of AKI as follows: for mild AKI the adjusted HR (aHR) was 2.0 (95% CI 1.4–2.8), for moderate AKI the aHR was 3.3 (95% CI 1.7–6.2), and for severe AKI the aHR was 28.2 (95% CI 21.1–37.5) [120]. Non-recovery from AKI, admission to hospital, the underlying cause of AKI, and age were determinant factors for the long-term outcome [118]. 

The evidence on the long-term effect of PR-AKI and the development of CKD is, however, limited. Kidney damage (isolated or co-existent glomerular, tubules, renal vasculature lesions) may be the common link of the complex relationship between pre-eclampsia, AKI, and CKD. Systematic reviews of mainly retrospective studies show that pre-eclampsia is associated with increased risk of albuminuria and end-stage renal diseases [3,121,122]. The long-term outcomes of PR-AKI from other aetiologies except pre-eclampsia and intrinsic kidney diseases have not been thoroughly studied. A recent retrospective study demonstrates that women with a past episode of AKI, despite returning to normal renal function before pregnancy, do experience adverse outcomes in pregnancy such as increased risk of pre-eclampsia (adjusted odds ratio aOR 5.9) and small for gestational age births (aOR 2.4) [123].

Taken together, the data from non-pregnant patients and the evidence from the pre-eclampsia-AKI-CKD studies, it can be inferred that AKI episodes in pregnancy may increase the risk of kidney damage in long-term, although the magnitude of this association is not known. Not all data, however, are supporting the progressive features of AKI in pregnancy, with some pleading for the rejuvenating effect of pregnancy on pathological processes in the organism, thus the adverse effects are mitigated by the physiological changes of pregnancy. A recent study provided evidence to support that pregnancy-associated changes upregulate regeneration- and proliferation-associated pathways, thus activating cell-protective signalling pathways that render kidneys more tolerant of acute kidney injury during pregnancy [124].

As the number of women survivors of PR-AKI increases and the evidence of AKI progression is mounting, a better understanding of the clinical outcomes after AKI is needed in order to identify those women at higher risk for adverse consequences and develop strategies to optimize their care. Although prenatal consultations aim to identify potential risk factors that may render the pregnancy high-risk, previous AKI or PR-AKI is rarely drawn attention to as an individual risk, commonly concealed under the umbrella of other obstetrical risk factors. In spite of the mounting evidence that links pre-eclampsia, AKI, and CKD, longitudinal follow up of these patients is not common practice. Publications promoting best practice in long-term follow-up and postpartum management of patients with pre-eclampsia and kidney disease are starting to emerge [5,125]. In clinical practice we arrange for the mothers to have their renal function, urine protein, and blood pressure checked after the postpartum period, 6–12 months after delivery.

## 8. Conclusions

After a reported decrease in the incidence of PR-AKI, the epidemiologists have seen a trend in the increase of PR-AKI over the past years. Foetal and maternal morbidity and mortality associated with this pregnancy complication remains high, even in developed countries. Hypertensive complications of pregnancy are the leading cause of PR-AKI in developed countries, whereas sepsis (post-abortion of puerperal) and haemorrhage, especially in postpartum, are the predominant causes in developing countries. Management of PR-AKI remains clinically challenging because of changes in the risk and causative factors as well as the characteristics of the childbearing population. Breakthroughs in the understanding of the pathophysiological mechanisms of PR-AKI and new therapies available as well as implementation of programs dedicated to maternal health may lead to better management of these patients and improvement in their health outcomes.

## Figures and Tables

**Table 1:** Changes in renal haemodynamics variables, glomerular filtration rate (GFR), effective renal plasma flow (ERPF), and filtration fraction (FF) during gestation. The values represent a percentage increase from the baseline, non-pregnant values. Data retrieved from references [17,18].

% Change	Pregnant			Postnatal	
	1–20 weeks	20–30 weeks	30–40 weeks	1–6 weeks	>6 weeks
Odutayo and Hladunewich [17]					
GFR	37.13	38.38	39.46	24.9	−0.91
ERPF	41.18	29.44	10.37	−5.13	−7.49
FF	−1.89	10.68	29.26	24.8	−1.59
Davison and Dunlop [18]					
GFR	48.9	45.8	51.0	-	-
ERPF	67.8	64.9	44.1	-	-
FF	−10.9	−10.9	5.8	-	-

GFR, glomerular filtration rate (mL/min); ERPF, effective renal plasma flow (mL/min); FF, filtration fraction (%).

**Table 2:** Laboratory values of renal function in pregnancy. Adapted from references [20,21,22,23]. Pregnant values of each variable are means of the variable values measured throughout pregnancy.

Renal Variable	Non-Pregnant Values	Pregnant Values	Values in Pregnancy that Require Further Investigation
Glomerular filtration rate (GFR) (mL/min)	106–132	130–180	<115
Effective renal plasma flow (ERPF) (mL/min)	492–696	630–1030	<590
Filtration Fraction (FF) (%)	16.9–24.7	15.4–22.8	<14.0
Serum Sodium (mEq/L)	136–146	133–148	<128
Serum Potassium (mEq/L)	3.5–5.0	3.3–5.0	>5.1
Serum Chloride (mEq/L)	102–109	97–109	>110
Serum Bicarbonate (mEq/L)	27–28	20–22	<20
Plasma osmolality (mOsm/kg H_2_O)	275–295	276–289	>290
pH (arterial)	7.35–7.45	7.40–7.45	<7.36; >7.45
Plasma urate (mg/dL)	4–6	2.5–4	>5.8
Plasma Creatinine (mg/dL, µmol/L)	0.51–1.02; (45–90)	0.59–0.87; (52–77)	>0.87 (77)
Creatinine clearance (mL/min)	91–130	110–150	<90
Blood urea nitrogen (mg/dL)	13 ± 3	8.7 ± 1.5	>14
Urinary glucose (mg/24 h)	20–100	>100	-
Urinary protein (mg/24 h)	<100–150	<250–300	>300
Urinary amino acids (g/24 h)	-	≤2	>2

**Table 3:** Risk, injury, failure, loss (RIFLE) system classification for acute kidney injury (AKI) and the modifications of the AKI classification criteria proposed by the Acute Kidney Injury Network (AKIN) network (adapted from references [16,33,36].

AKI Classification Systems					
RIFLE Criteria for Classification/Staging AKI			AKIN Criteria for Classification/Staging AKI		
Stage	GFR Criteria	Urine Output Criteria	Stage	Serum Creatinine Criteria	Urine Output Criteria
**Risk**	Increase in SCr ×1.5 or Decrease in GFR > 25%	UO < 0.5 mL/kg/h × 6 h	Stage 1	Increase in SCr ≥ 0.3 mg/dL or Increase SCr ≥ 1.5–2.0 ×	UO < 0.5 mL/kg/h × 6 h
**Injury**	Increase in SCr ×2.0 or Decrease in GFR >50%	UO < 0.5mL/kg/h × 12 h	Stage 2	Increase in SCr > 2.0–3.0 ×	UO < 0.5 mL/kg/h × 12 h
**Failure**	Increase in SCr × 3.0 or Decrease in GFR >75% or SCr >4.0 mg/dL (acute increase ≥ 0.5 mg/dL)	UO < 0.3mL/kg/h × 24 h or anuria for 12 h	Stage 3	Increase in SCr > 3 × or Increase of SCr to ≥4.0 mg/dL with an acute increase of at least 0.5 mg/dL	UO < 0.3 mL/kg/h × 24 h or anuria for 12 h
**Loss**	Persistent ARF: Complete loss of kidney function for >4 weeks			Patients who receive renal replacement therapy (RRT) are considered to have met the criteria for stage 3 irrespective of the stage they were in at the time of commencement of RRT.	
**ESKD**	End-stage kidney disease for >3 months				

Abbreviations: RIFLE, acronym for Risk, Injury, Failure, Loss, ESKD; AKI, acute kidney injury; GFR, glomerular filtration rate, UO, urine output; SCr, serum creatinine; ESRD, end-stage renal disease; ARF, acute renal failure.

**Table 4:** AKI staging according to Kidney Disease: Improving Global Outcomes (KDIGO) criteria (adapted from reference [36]).

AKI Classification Systems: KDIGO Criteria		
Stage	Serum Creatinine Criteria	Urine Output Criteria
**Stage 1**	Increase in SCr × 1.5–1.9 or Increase in SCr ≥ 0.3 mg/dL	UO < 0.5 mL/kg/h × 6–12 h
**Stage 2**	SCr ≥ 2.0–2.9 times baseline	UO < 0.5 mL/kg/h ≥ 12 h
**Stage 3**	Increase SCr ≥ 3.0 × or Increase in SCr to ≥ 4.0 mg/dL or Initiation of renal replacement therapy (RRT) or In patients < 18 years, decrease in eGFR to <35 mL/min per 1.73 m^2^	UO < 0.3 mL/kg/h × ≥24 h or Anuria for ≥ 12 h

Abbreviations: SCr, serum creatinine; OU, urine output; eGFR, estimated glomerular filtration rate.

**Table 5:** Causes of acute kidney injury in pregnancy classified by the time of occurrence.

Pre-Renal	Intrinsic Renal	Post-Renal
**Early Pregnancy**		
Bleeding—miscarriage	Anticardiolipin antibody syndrome	Renal stones
Hyperemesis gravidarum	Sepsis (i.e., septic abortion)	Ureteral obstruction
Ovarian hyperstimulation syndrome	Autoimmune disease	
Ectopic pregnancy	Glomerulonephritis, interstitial nephritis, lupus nephritis	
	CKD progression	
**Late Pregnancy**		
Bleeding—second-trimester miscarriage, placenta praevia, placental abruption	Severe pre-eclampsia, HELLP	Polyhydramnios
	Acute fatty liver of pregnancy	Multifetal gestation
	HUS/TTP	Large uterine fibroids
	Pyelonephritis	Ureteral obstruction
	Chorioamnionitis	Renal stones
	CKD Progression	
	Glomerulonephritis, interstitial nephritis, lupus nephritis	
**Postpartum**		
Bleeding—uterine atonia, uterine rupture, obstetrical trauma (vulvo-vaginal and perineal tears and lacerations)	Severe pre-eclampsia, HELLP	Renal stones
	HUS	
	Puerperal sepsis	
	Glomerulonephritis, interstitial nephritis, lupus nephritis	
	Nephrotoxic drugs (NSAIDS, antibiotics, proton-pump inhibitors, H2 antagonists)	
	CKD Progression	

Abbreviations: HELLP, haemolysis, elevated liver enzymes and low platelet count; HUS, haemolytic uremic syndrome; NSAIDs, non-steroidal anti-inflammatory drugs; TTP, thrombotic thrombocytopenic purpura; CKD, chronic kidney disease.
